# Preoperative leukocytosis and the resection severity index are independent risk factors for survival in patients with intrahepatic cholangiocarcinoma

**DOI:** 10.1007/s00423-020-01962-4

**Published:** 2020-08-19

**Authors:** Oliver Beetz, Clara A. Weigle, Sebastian Cammann, Florian W. R. Vondran, Kai Timrott, Ulf Kulik, Hüseyin Bektas, Jürgen Klempnauer, Moritz Kleine, Felix Oldhafer

**Affiliations:** 1 Department of General, Visceral and Transplant Surgery, Hannover Medical School, Carl-Neuberg-Strasse 1, 30625 Hannover, Germany; 2 Department of General, Visceral and Oncological Surgery, Hospital Group Gesundheit Nord, Bremen, Germany

**Keywords:** Intrahepatic cholangiocarcinoma, Resection severity index, Extended surgery, Leukocytosis

## Abstract

**Purpose:**

The incidence of intrahepatic cholangiocarcinoma is increasing worldwide. Despite advances in surgical and non-surgical treatment, reported outcomes are still poor and surgical resection remains to be the only chance for long-term survival of affected patients. The identification and validation of prognostic factors and scores, such as the recently introduced resection severity index, for postoperative morbidity and mortality are essential to facilitate optimal therapeutic regimens.

**Methods:**

This is a retrospective analysis of 269 patients undergoing resection of histologically confirmed intrahepatic cholangiocarcinoma between February 1996 and September 2018 at a tertiary referral center for hepatobiliary surgery. Regression analyses were performed to evaluate potential prognostic factors, including the resection severity index.

**Results:**

Median postoperative follow-up time was 22.93 (0.10–234.39) months. Severe postoperative complications (≥ Clavien-Dindo grade III) were observed in 94 (34.9%) patients. The body mass index (*p* = 0.035), the resection severity index (ASAT in U/l divided by Quick in % multiplied by the extent of liver resection graded in points; *p* = 0.006), additional hilar bile duct resection (*p* = 0.005), and number of packed red blood cells transfused during operation (*p* = 0.036) were independent risk factors for the onset of severe postoperative complications. Median Kaplan-Meier survival after resection was 27.63 months. Preoperative leukocytosis (*p* = 0.003), the resection severity index (*p* = 0.005), multivisceral resection (*p* = 0.001), and T stage ≥ 3 (*p* = 0.013) were identified as independent risk factors for survival.

**Conclusion:**

Preoperative leukocytosis and the resection severity index are useful variables for preoperative risk stratification since they were identified as significant predictors for postoperative morbidity and mortality, respectively.

## Introduction

Intrahepatic cholangiocarcinoma (ICC) is a malignant disease of the intrahepatic bile ducts. Although only accounting for 10 to 20% of primary hepatic malignancies, incidence and mortality rates are continuously increasing world-wide [1]. Recent epidemiological data from Germany has shown incidence and mortality rates of 1 to 2 cases per 100,000 [2].

Despite the introduction of more effective chemotherapy regimens in the past, the prognosis of a majority of patients with unresectable ICC is dismal, with reported median survival times of approximately 12 months [3, 4]. Therefore, local ablative procedures, such as radiofrequency ablation, transarterial chemoembolization, or selective internal radiation therapy and even liver transplantation are increasingly under (re) evaluation [5–9].

Complete surgical resection is still regarded as only chance for long-term survival of affected patients; however, the reported survival after resection remains poor with 5-year survival rates ranging between 20 and 35% [10].

In light of the above-mentioned epidemiologic trends and developing therapeutic alternatives, identification of prognostic factors for morbidity and mortality after surgical resection of ICC, especially in cases of advanced disease, is essential to determine optimal treatment strategies.

Recently, we have introduced the resection severity index (RSI) reflecting the degree of liver function and liver cell damage, as well as the extent of hepatic resection, as an independent risk factor for survival in patients with hepatocellular carcinoma and colorectal liver metastases undergoing surgery [11, 12].

The aim of this study was to evaluate the influence of the RSI among other factors on the postoperative morbidity and mortality of patients with ICC undergoing hepatic resection.

## Material and methods

### Study design and patient cohort

This is a retrospective analysis of 269 patients with ICC undergoing surgery between February 1996 and September 2018 at the Department of General, Visceral and Transplant Surgery, Hannover Medical School, Germany.

### Inclusion and exclusion criteria

Included were all patients with intraoperatively and histologically confirmed ICC receiving hepatic resection in curative intent older than 18 years of age. Patients with hilar cholangiocarcinoma (defined by the localization of the main tumor mass) were explicitly excluded from our study

Patients undergoing surgery for tumor recurrence were excluded from the study. Four patients were lost to follow-up immediately after discharge and were therefore excluded from further survival analysis. No further exclusion criteria were defined.

### Definition of variables

Preoperative leukocytosis was defined as a leukocyte concentration of more than 11,000 per μl [13]. Preoperative anemia was defined as hemoglobin concentrations lower than 11.5 g/dl in female patients and lower than 13.5 g/dl in male patients, as recently suggested [14].

The resection severity index (RSI) was initially developed as an interaction variable in a multivariable regression model in patients with hepatocellular carcinoma undergoing surgical resection and is defined as ASAT in U/l divided by Quick in % multiplied by the extent of liver resection graded in points (Table 1) [11]. The theoretical background of this mathematical formula is an attempt to quantify the degree of liver damage (ASAT), functional liver capacity (Quick), and the expected or actual extent of liver resection (graded in points) and thus the (future) functional liver remnant. For regression analysis, the RSI was used as continuous variable, whereas for Log-rank analysis, three tertiles were computed (low, intermediate, and high).

**Table 1:** Descriptive statistics of the study cohort undergoing resection for intrahepatic cholangiocarcinoma

Variables			*n*_abs_ (*n*_%_)	Mean; median (range)	Missing values *n* (%)
Biometrics	Male gender		134 (49.8)		0 (0)
	Female gender		135 (50.2)		
	Age (in years)			61.11; 62 (24–83)	0 (0)
	Body mass index (in kg/m^2^)			25.74; 25.20 (16.36–55.36)	5 (1.9)
Preoperative laboratory results	Hemoglobin (in g/dl)			13.14; 13.30 (8.2–17.2)	1 (0.4)
	Anemia		78 (29.0)		1 (0.4)
	Leukocytes (in 10^3^/μl)			8.32; 7.7 (1.7–24.1)	1 (0.4)
	Leukocytosis		39 (14.5)		1 (0.4)
	Platelets (in 10^3^/μl)			281; 254 (69–902)	2 (0.7)
	Quick (in %)			97.79; 99.5 (46–147)	3 (1.1)
	ASAT (in U/l)			38.8; 30.5 (4–304)	7 (2.6)
	ALAT (in U/l)			41.0; 26 (5–543)	49 (18.2)
	Bilirubin (in μmol/l)			20.6; 9 (3–445)	10 (3.7)
	Creatinine (in μmol/l)			69.27; 66 (39–165)	6 (2.2)
Surgical details	Major hepatectomy		224 (83.3)		0 (0)
	Extended hepatectomy		95 (35.3)		
	Resection points	1 (atypical)	17 (6.3)		0 (0)
		2 (segmental)	31 (11.5)		
		3 (left hemihepatectomy)	85 (31.6)		
		4 (right hemihepatectomy)	41 (15.2)		
		5 (extended left)	31 (11.5)		
		6 (extended right)	64 (23.8)		
	RSI (ASAT ÷ Quick × Resection points)			1.69; 1.04 (0.13–33.78)	9 (3.3)
	Hilar bile duct resection		51 (19.0)		0 (0)
	Vascular resection		10 (3.7)		
	Multivisceral resection		11 (4.1)		
	Operation time (in min)			203.77; 190 (67–780)	4 (1.5)
	Pringle maneuver		208 (77.3)		26 (9.7)
	Pringle maneuver (in min)			23.19; 22 (0–110)	
	Intraoperative PRBC		130 (48.3)		6 (2.2)
	Intraoperative PRBC (*n*)			2.03; 0 (0–17)	
Postoperative pathological results	T staging	Tumor size (in cm)		7.55; 7.0 (0.5–21.0)	2 (0.7)
		Vascular invasion	59 (21.9)		109 (40.5)
		Multifocal	98 (36.4)		0 (0)
		1a	22 (8.2)		44 (16.4)
		1b	45 (16.7)		
		2	99 (36.8)		
		3	16 (5.9)		
		4	43 (16.0)		
		≥ 3	59 (21.9)		1 (0.4)
	Lymph node status	Lymphadenectomy	172 (63.9)		0 (0)
		Lymph nodes (n total)		4.98; 3 (1–23)	98 (36.4)
		Lymph nodes (n positive)		1.13; 0 (0–11)	98 (36.4)
		N 1 stage	76 (28.3)		96 (35.7)
	M 1 stage		7 (2.6)		0 (0.0)
	Grading	1	3 (1.1)		5 (1.9)
		1–2	2 (0.7)		
		2	183 (68.0)		
		2–3	12 (4.5)		
		3	64 (23.8)		
	Resection margin	0	223 (82.9)		3 (1.1)
		1	37 (13.8)		
		x	6 (2.2)		
	AJCC/UICC classification (8^th^ ed.)	Ia	7 (2.6)		95 (35.3)
		Ib	21 (7.8)		
		II	34 (12.6)		
		IIIa	4 (1.5)		
		IIIb	101 (37.5)		
		IV	7 (2.6)		
		≥ IIIa	118 (43.9)		77 (28.6)
	Steatosis	Mild	76 (28.3)		9 (3.3)
		Moderate	15 (5.6)		
		Severe	0 (0.0)		
	Fibrosis		66 (24.5)		0 (0.0)
	Cirrhosis		11 (4.1)		
	Cholestasis		37 (13.8)		
	Cholangitis		17 (6.3)		

Clinical, surgical, and histopathological data of the 269 patients undergoing surgery for intrahepatic cholangiocarcinoma

*ASAT* aspartate aminotransferase, *ALAT* alanine aminotransferase, *RSI* resection severity index, *PRBC* packed red blood cells, *AJCC* American Joint Committee on Cancer, *UICC* Union for International Cancer Control

Major hepatectomies were defined as resections of three or more liver segments, whereas extended hepatectomies were defined as resection of five or more segments, based on the Brisbane classification [15]. Vascular resections were defined as additional partial resection of the vena cava inferior or the portal vein. Multivisceral resections were defined as additional resections of extrahepatic tissue (excluding hilar bile ducts and large vessels) due to suspected infiltration.

Postoperative complications were graded according to the Clavien-Dindo classification ranging from grade 0 (no complications), grade I (minor deviations), grade II (requiring pharmacologic treatment), grade III (requiring interventions), grade IV (life-threatening) to grade V (death) [16]. Severe complications were defined as complications ≥ grade III.

Additionally, typical posthepatectomy complications (hemorrhage, liver failure, or biliary leakage) requiring invasive treatment and surgical revision, respectively, (classified as Grade C complications by the International Study Group of Liver Surgery (ISGLS)) were evaluated [17].

For classification of ICC, the AJCC/UICC 8^th^ edition was applied [18]. Of note, to avoid retrospective misclassification, patients were only classified if pathological parameters available allowed for distinct allocation.

Liver specimens were further reviewed for steatosis (graded into mild (5–33%), moderate (34–66%), or severe (> 66%)), fibrosis, and cirrhosis as well as for cholestasis and cholangitis [19].

Three approximately equally long time periods were defined to analyze if not further specifiable adjustments or improvements in perioperative care and surgical strategies had impact on postoperative survival.

Follow-up time was defined as time between date of surgery and date of last contact or death, respectively. Survival times are reported as the Kaplan-Meier median estimates.

### Study endpoints

Primary endpoints were the incidence of severe postoperative complications and overall survival after resection of ICC.

### Statistical analysis

Mean and median values were compared with Student’s *t* test in case of normal distribution or the Mann-Whitney *U* test. The distribution of categorical variables between groups was compared with the chi-squared and the Fisher’s exact test.

Risk factors for the incidence of severe postoperative complications were analyzed with univariable binary logistic regression analysis. Independent risk factors were identified by purposeful selection of variables with a rate of missing values of < 10% and *p* values in univariable logistic regression of < 0.300 and consecutive stepwise forward selection.

The identification of risk factors for postoperative survival was achieved by univariable and multivariable Cox regression analysis, as described above. Kaplan-Meier analyses including log-rank tests were performed where appropriate.

Statistical significance was set at a *p* value of < 0.050 and is shown bold (tables) or marked with an asterisk (figures).

The collected data was implemented and analyzed using SPSS statistical software (version 26; SPSS Inc.; IBM Corporation, Armonk, NY, USA). Figures were created with GraphPad Prism (version 8.3.0 for Windows, GraphPad Software, La Jolla, CA, USA).

## Results

### Preoperative course

The median age of patients undergoing hepatic resection for ICC was 62 (24–83) years. An equal gender distribution was observed among the included patients.

**Fig. 1:**
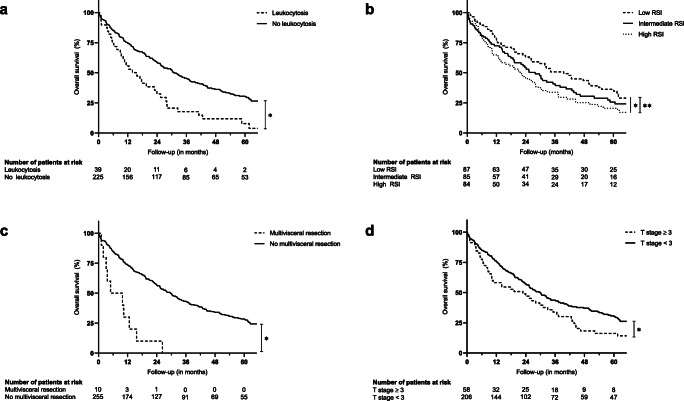
Kaplan-Meier survival after resection of intrahepatic cholangiocarcinoma for variables identified as independent significant risk factors. **a** Preoperative leukocytosis (*p = 0.001). **b** Low, intermediate, or high RSI (general trend *p = 0.040; low versus high **p = 0.009). **c** Multivisceral resection (*p < 0.001). **d** T stage ≥ 3 (*p = 0.021) (statistical significance (*p* < 0.050) is indicated with an asterisk)

Upon admission, standardized preoperative laboratory testing revealed anemia in 78 (29.0%) patients, and leukocytosis in 39 (14.5%) patients. Whereas anemia resulted in a non-significant lower median survival (25.23 versus 30.33 months; *p* = 0.052), preoperative leukocytosis was associated with a significantly inferior survival after hepatic resection (15.71 versus 31.87 months; *p* = 0.001; Fig. 1a). Of note, median serum bilirubin concentration was not significantly elevated in case of preoperative leukocytosis (10 versus 9 μmol/l; *p* = 0.814).

Further information on biometrical and laboratory data is provided in Table 1.

### Hepatic resection

Atypical or segmental resections were carried out in 48 (17.8%) patients. Left or right hemihepatectomy was performed in 126 (46.8%) patients. Ninety-five (35.3%) patients received extended left or right hemihepatectomy. None of the operations was performed minimally invasive.

The RSI was calculated for each patient as described above, resulting in a median value of 1.04 (0.13–33.78), and three tertiles: low (≤ 0.71; 87 patients), intermediate (0.72–1.63; 86 patients), and high (≥ 1.64; 87 patients). Further analyses revealed a significant influence of RSI classification on postoperative survival (39.62 > 27.93 > 22.28 months; general trend: *p* = 0.040; low versus high *p* = 0.009; low versus intermediate: *p* = 0.313; intermediate versus high: *p* = 0.164; Fig. 1b).

Major hepatectomies were performed in 224 (83.3%) patients. Of these, 95 (42.4%) cases were graded as extended resections.

Additional hilar bile duct resection was carried out in 51 (19.0%) patients and was associated with significantly inferior postoperative survival (14.88 versus 28.16 months; *p* = 0.012).

Ten (3.7%) patients underwent additional vascular resection of the inferior vena cava or portal vein and showed significantly inferior postoperative survival (5.62 versus 29.50 months; *p* = 0.027).

Multivisceral resections, including (partial) resections of the diaphragm, peritoneum, pericardium, stomach, greater omentum, small intestine, pancreas, and kidney were performed in 11 (4.1%) patients due to intraoperatively suspected continuous or distant infiltration and were associated with significantly inferior survival (4.96 versus 29.70 months; *p* < 0.001; Fig. 1c).

A total of 208 (77.3%) patients underwent the Pringle maneuver during hepatic resection. Clamping was performed intermittent: Hepatic perfusion was interrupted for a maximum of 10 min before unclamping the hepatoduodenal ligament for a minimum of 5 min. The values reported in Table 1 refer to the total amount of interrupted hepatic blood for each patient.

Intraoperative blood transfusion was performed in 130 (48.3%) patients with a median of 3 (1–17) units of packed red blood cells (PRBC) and was associated with significantly inferior postoperative survival (22.93 versus 31.87 months; *p* = 0.011).

Surgical details are summarized in Table 1.

### Histopathological results

Advanced local tumor growth (defined as T stage ≥ 3) was observed in 59 (21.9%) patients and significantly influenced patient survival (22.83 versus 29.50 months; *p* = 0.021; Fig. 1d). Tumor multifocality (observed in 98 (36.4%) patients) was associated with higher RSI values (mean: 1.89 versus 1.57; median: 1.20 versus 0.86; *p* = 0.001).

Lymphadenectomy was performed in 172 (63.9%) patients, verifying regional lymph node metastases (N1) in 76 (28.3%) patients. Both variables (lymphadenectomy and positive nodal status) were associated with significantly inferior survival (25.23 versus 38.11 months; *p* = 0.007 and 14.36 versus 32.63 months; *p* < 0.001, respectively). Of note, we observed a non-significant trend towards increased rates of lymphadenectomy during the analyzed time period (1996–2003: 60.0%, 2004–2011: 63.5%, 2012–2018: 68.2%; *p* = 0.541).

Distant metastases were observed in 7 (2.6%) patients with peritoneal metastases being observed most frequently (four patients) followed by metastases in the greater omentum, the kidney, and the mediastinal lymph nodes (one patient each). Distant metastases were associated with significantly inferior survival (4.96 versus 28.03 months; *p* < 0.001).

Positive resection margins (R1) were observed in 37 (13.8%) patients. Further analyses did not reveal significant influence of R1 status on postoperative median survival (20.93 versus 29.50 months; *p* = 0.376).

Due to the low rate of lymphadenectomy and missing information on vascular invasion, distinct allocation into the AJCC/UICC stages was only possible for 174 (64.7%) patients. Advanced AJCC/UICC stages of ≥ IIIa were observed in 118 (43.9%) patients.

The rate of histological evidence for cholestasis (37 (13.8%) patients) or cholangitis (17 (6.3%) patients) was not significantly increased in patients with preoperative leukocytosis (20.5% versus 12.7%, *p* = 0.144 and 12.8% versus 5.2%, *p* = 0.082, respectively).

The histopathological results are summarized in Table 1.

### Postoperative course and outcome

Severe postoperative complications after hepatic resection were observed in 94 (34.9%) patients. Eighteen (6.7%) patients died in the postoperative course, mainly due to posthepatectomy liver failure (seven patients). Further lethal complications were biliary leakage (four patients), cardiac failure (two patients), pneumonia (two patients), severe bleeding, portal vein thrombosis, and mesenteric ischemia (one patient each) ultimately leading to multiple organ failure.

Patients were followed up after hepatic resection with a median of 22.93 (0.10–234.39) months.

Estimated median postoperative survival was 27.63 months. The 1-, 3-, and 5-year survival rates were 71.9%, 41.5%, and 27.4%, respectively.

Of note, the postoperative survival did not significantly alter over the course of time (1996–2003 (80 patients): 26.32 months, 2004–2011 (104 patients): 27.93 months, 2012–2018 (85 patients): 26.78 months; *p* = 0.776).

Table 2 summarizes the postoperative course and outcome after hepatic resection.

**Table 2:** Postoperative outcome of the study cohort after resection of intrahepatic cholangiocarcinoma

Variables		*n*_abs_ (*n*_%_)	Mean; median (range)	Missing values *n* (%)
Postoperative PRBC		71 (26.4)		10 (3.7)
Postoperative PRBC (*n*)			1.38; 0 (0–29)	
Intensive care unit stay (in days)			4.36; 2 (0–91)	0 (0)
Hospital stay (in days)			23.18; 20 (4–95)	
Postoperative complications (classified by Clavien-Dindo)	0	59 (21.9)		2 (0.7)
	I	37 (13.8)		
	II	77 (28.6)		
	IIIa	34 (12.6)		
	IIIb	37 (13.8)		
	IVa	5 (1.9)		
	V	18 (6.7)		
	Severe complications (≥ IIIa)	94 (34.9)		
Postoperative liver-specific complications Grade C (classified by ISGLS)	Hemorrhage	8 (3.0)		2 (0.7)
	Liver failure	13 (4.8)		
	Biliary leakage	13 (4.8)		
30-day mortality		11 (4.1)		4 (1.5)
90-day mortality		21 (7.8)		5 (1.9)
Follow-up time in months			38.75; 22.93 (0.10–234.39)	4 (1.5)
Survival in months (Kaplan-Meier)			52.61; 27.63 (n.a.)	
1-year survival (Kaplan-Meier)		n.a. (71.9)		
3-year survival (Kaplan-Meier)		n.a. (41.5)		
5-year survival (Kaplan-Meier)		n.a. (27.4)		
Deceased at time of analysis		207 (77.0)		

Postoperative outcome of the 269 patients undergoing surgery for intrahepatic cholangiocarcinoma

*PRBC* packed red blood cells, *n.a.* not applicable

### Identification of independent risk factors for postoperative morbidity and mortality

The results from univariable analysis evaluating risk factors for the incidence of severe postoperative complications are displayed in Table 3.

**Table 3:** Logistic regression analysis for identification of risk factors for the incidence of severe postoperative complications after hepatic resection

Variables		Univariable analysis			Multivariable analysis		
		OR	CI-95%	*p* value	OR	CI-95%	*p* value
Biometrics	Male gender	1.127	(0.682–1.863)	0.640			
	Age (in years)	0.991	(0.969–1.014)	0.447			
	Body mass index (kg/m^2^)	1.083	(1.022–1.148)	*0.007*	1.072	(1.005–1.143)	**0.035**
Preoperative laboratoryresults	Hemoglobin (in g/dl)	0.945	(0.817–1.092)	0.441			
	Anemia	1.211	(0.701–2.092)	0.493			
	Leucocytes (in 10^3^/μl)	1.031	(0.952–1.116)	0.456			
	Leukocytosis	1.703	(0.857–3.386)	0.129			
	Platelets (in 10^3^/μl)	1.000	(0.998–1.002)	0.911			
	Quick (in %)	0.982	(0.966–0.999)	*0.041*			
	ASAT (in U/l)	1.011	(1.003–1.019)	*0.008*			
	ALAT (in U/l)	1.005	(1.000–1.011)	0.071			
	Bilirubin (in μmol/l)	1.007	(1.000–1.014)	*0.036*			
	Creatinine (in μmol/l)	1.000	(0.986–1.015)	0.958			
Surgical details	Major hepatectomy	3.498	(1.495–8.185)	*0.004*			
	Extended hepatectomy	1.418	(0.842–2.386)	0.189			
	Resection points (continuous)	1.260	(1.067–1.490)	*0.007*			
	Resection points 1	0.229	(0.051–1.024)	0.054			
	Resection points 2	0.500	(0.207–1.207)	0.123			
	Resection points 3	0.758	(0.437–1.316)	0.325			
	Resection points 4	2.201	(1.123–4.313)	*0.022*			
	Resection points 5	1.014	(0.463–2.218)	0.973			
	Resection points 6	1.534	(0.860–2.734)	0.147			
	RSI	1.409	(1.157–1.717)	*0.001*	1.335	(1.084–1.643)	**0.006**
	Hilar bile duct resection	3.393	(1.808–6.367)	*< 0.001*	2.825	(1.358–5.876)	**0.005**
	Vascular resection	2.881	(0.792–10.477)	0.108			
	Multivisceral resection	1.564	(0.464–5.267)	0.471			
	Operation time (in min)	1.005	(1.002–1.008)	*0.001*			
	Pringle maneuver	0.909	(0.433–1.912)	0.802			
	Pringle maneuver (in min)	1.006	(0.990–1.023)	0.436			
	Intraoperative PRBC	1.681	(1.007–2.805)	*0.047*			
	Intraoperative PRBC (*n*)	1.156	(1.054–1.267)	*0.002*	1.116	(1.007–1.237)	**0.036**
Postoperativepathological results	Tumor size (in cm)	1.028	(0.964–1.097)	0.396			
	Vascular invasion	1.861	(0.963–3.597)	0.065			
	Multifocal	1.616	(0.963–2.711)	0.069			
	T stage ≥ 3	2.200	(1.217–3.978)	*0.009*			
	Lymphadenectomy	1.110	(0.655–1.880)	0.699			
	Lymph nodes (*n* total)	1.056	(0.986–1.131)	0.122			
	Lymph nodes (*n* positive)	1.138	(0.974–1.329)	0.104			
	N 1 stage	1.143	(0.613–2.132)	0.674			
	M 1 stage	0.299	(0.035–2.524)	0.267			
	Grading > 2	1.537	(0.887–2.665)	0.126			
	Positive resection margin (R1)	1.234	(0.600–2.538)	0.568			
	AJCC/UICC8 ≥ IIIa	1.555	(0.845–2.859)	0.156			
	Steatosis	1.273	(0.748–2.166)	0.374			
	Fibrosis	1.166	(0.656–2.076)	0.600			
	Cirrhosis	1.564	(0.464–5.267)	0.471			
	Cholestasis	0.996	(0.482–2.062)	0.992			
	Cholangitis	1.695	(0.631–4.550)	0.295			

Results of the binary logistic regression analysis for identification of risk factors for the incidence of severe postoperative complications after resection of intrahepatic cholangiocarcinoma. Bold values indicate statistical significance (*p* < 0.050) in univariable or multivariable analysis

*OR* odds ratio, *CI* confidence interval, *ASAT* aspartate aminotransferase, *ALAT* alanine aminotransferase, *RSI* resection severity index, *PRBC* Packed red blood cells, *AJCC* American Joint Committee on Cancer, *UICC* Union for International Cancer Control

Multivariable analysis identified the body mass index (OR: 1.072; CI-95%: 1.005–1.143; *p* = 0.035), the RSI (OR: 1.335; CI-95%: 1.084–1.643; *p* = 0.006), hilar bile duct resection (OR: 2.825; CI-95%: 1.358–5.876; *p* = 0.005), and number of PRBC transfused intraoperatively (OR: 1.116; CI-95%: 1.007–1.237; *p* = 0.036) as independent significant risk factors for severe postoperative complications (Table 3).

The results from univariable analysis evaluating risk factors for postoperative survival are displayed in Table 4.

**Table 4:** Cox regression analysis for identification of risk factors for survival after hepatic resection of intrahepatic cholangiocarcinoma

Variables		Univariable analysis			Multivariable analysis		
		HR	CI-95%	*p* value	HR	CI-95%	*p* value
Biometrics	Male gender	0.962	(0.732–1.264)	0.783			
	Age (in years)	0.997	(0.985–1.011)	0.703			
	Body mass index (kg/m^2^)	1.019	(0.988–1.050)	0.237			
Preoperative laboratory results	Hemoglobin (in g/dl)	0.915	(0.844–0.992)	*0.032*			
	Anemia	1.341	(0.997–1.803)	0.053			
	Leukocytes (in 10^3^/μl)	1.088	(1.044–1.133)	*< 0.001*			
	Leukocytosis	1.907	(1.313–2.771)	*0.001*	1.857	(1.232–2.799)	**0.003**
	Platelets (in 10^3^/μl)	1.001	(1.000–1.002)	0.061			
	Quick (in %)	0.984	(0.974–0.994)	*0.003*			
	ASAT (in U/l)	1.005	(1.001–1.009)	*0.024*			
	ALAT (in U/l)	1.002	(0.999–1.005)	0.290			
	Bilirubin (in μmol/l)	1.004	(1.002–1.007)	*0.002*			
	Creatinine (in μmol/l)	1.003	(0.995–1.011)	0.514			
Surgical details	Major hepatectomy	1.253	(0.846–1.857)	0.261			
	Extended hepatectomy	1.146	(0.865–1.520)	0.342			
	Resection points (continuous)	1.062	(0.972–1.159)	0.182			
	Resection points 1	0.705	(0.373–1.332)	0.281			
	Resection points 2	0.956	(0.608–1.503)	0.845			
	Resection points 3	0.847	(0.631–1.137)	0.270			
	Resection points 4	1.259	(0.865–1.832)	0.229			
	Resection points 5	1.219	(0.807–1.843)	0.346			
	Resection points 6	1.063	(0.776–1.457)	0.703			
	RSI	1.104	(1.051–1.161)	*< 0.001*	1.081	(1.024–1.141)	**0.005**
	Hilar bile duct resection	1.534	(1.096–2.146)	*0.013*			
	Vascular resection	2.105	(1.072–4.133)	*0.031*			
	Multivisceral resection	4.387	(2.280–8.440)	*< 0.001*	3.665	(1.751–7.671)	**0.001**
	Operation time (in min)	1.002	(1.001–1.004)	*< 0.001*			
	Pringle maneuver	0.815	(0.554–1.199)	0.300			
	Pringle maneuver (in min)	1.002	(0.992–1.011)	0.703			
	Intraoperative PRBC	1.427	(1.082–1.883)	*0.012*			
	Intraoperative PRBC (*n*)	1.086	(1.037–1.137)	*< 0.001*			
Postoperative pathological results	Tumor size (in cm)	1.024	(0.990–1.059)	0.169			
	Vascular invasion	2.201	(1.505–3.221)	*< 0.001*			
	Multifocal	1.497	(1.128–1.986)	*0.005*			
	T stage ≥ 3	1.452	(1.056–1.996)	*0.022*	1.532	(1.094–2.146)	**0.013**
	Lymphadenectomy	1.488	(1.113–1.991)	*0.007*			
	Lymph nodes (n total)	1.028	(0.994–1.063)	0.103			
	Lymph nodes (n positive)	1.105	(1.032–1.184)	*0.004*			
	N 1 stage	2.193	(1.544–3.116)	*< 0.001*			
	M 1 stage	4.384	(1.765–10.891)	*0.001*			
	Grading > 2	1.140	(0.848–1.533)	0.384			
	Positive resection margin (R 1)	1.192	(0.808–1.759)	0.377			
	AJCC/UICC8 ≥ IIIa	1.903	(1.355–2.673)	*< 0.001*			
	Steatosis	1.028	(0.763–1.386)	0.854			
	Fibrosis	1.008	(0.729–1.394)	0.962			
	Cirrhosis	1.282	(0.656–2.505)	0.467			
	Cholestasis	1.278	(0.861–1.897)	0.223			
	Cholangitis	1.220	(0.695–2.143)	0.488			

Results of the Cox regression analysis for identification of risk factors for survival after resection of intrahepatic cholangiocarcinoma. Bold values indicate statistical significance (*p* < 0.050) in univariable or multivariable analysis

*HR* hazard ratio, *CI* confidence interval, *ASAT* aspartate aminotransferase, *ALAT* alanine aminotransferase, *RSI* resection severity index, *PRBC* packed red blood cells, *AJCC* American Joint Committee on Cancer, *UICC* Union for International Cancer Control

Multivariable analysis identified preoperative leukocytosis (HR: 1.857; CI-95%: 1.232–2.799; *p* = 0.003), the RSI (HR: 1.081; CI-95%: 1.024–1.141; *p* = 0.005), multivisceral resection (HR: 3.665; CI-95%: 1.751–7.671; *p* = 0.001), and T stage ≥ 3 (HR: 1.532; CI-95%: 1.094–2.146; *p* = 0.013) as independent significant risk factors for postoperative survival (Table 4).

## Discussion

The prognosis of patients with ICC remains unsatisfying, despite efforts of improving surgical and non-surgical treatment in the past. Although the importance of surgical resection to achieve long-term survival is undisputed, not all patients benefit from hepatic resections. Especially in cases of advanced disease, poor survival rates were reported in previous publications of larger mono- and multicentric studies as confirmed in our present work [10, 14, 20]. With respect to the simultaneously reported considerable postoperative mortality of up to 10%, preoperative risk stratification is imperatively indicated [21].

Several studies have emphasized the prognostic value of inflammatory parameters and indices, such as elevated serum C-reactive protein levels or elevated neutrophil to lymphocyte ratios on postoperative survival in patients undergoing surgery for ICC [22–27]. To our knowledge, our study is the first to identify preoperative leukocytosis as an independent risk factor for survival. Although patients with high leukocyte concentrations did not show significantly elevated serum bilirubin concentration as a sign of obstructive cholestasis, concomitant elevation of serum gamma-glutamyltransferase and alkaline phosphatase, as well as C-reactive protein levels, was detected in most patients with preoperative leukocytosis prompting the assumption of underlying cholangitis as reason for our observations. However, only four patients exhibited typical clinical symptoms, such as fever, rigor, or jaundice and postoperative histological analysis did not reveal a statistically significant association between preoperative leukocytosis and the evidence of cholestasis or cholangitis. Furthermore, we did not observe an increase of severe or septic postoperative complications in the respective subgroup, and increased mortality was not limited to the immediate postoperative course but was observed throughout the entire follow-up. Of note, asymptomatic preoperative leukocytosis has been identified as a risk factor for an increase in morbidity and mortality in patients undergoing surgery for colorectal carcinoma. The authors state that apart from an inflammatory environment depending on the respective tumor immunogenicity, most commonly discussed as explanation for the findings, preoperative dehydration and malnutrition leading to leukocytosis could also be a reason for an inferior postoperative outcome [28]. In summary, the association between preoperative leukocytosis and the observed increase in mortality in our patients remains unclear and needs further evaluation in prospective clinical trials.

Prognostic stratification scores for survival after resection of different malignancies, including ICC, have been published in recent years to optimize perioperative decision making [29–32]. We have recently introduced the RSI, estimating the future functional liver remnant with respect to the preoperative liver function and the expected or actual extent of hepatic surgery, as an independent risk factor for survival after resection of hepatocellular carcinoma and colorectal liver metastases [11, 12]. In the current study, we were able to demonstrate the significance of the RSI as an independent risk factor for the onset of severe postoperative complications, as well as postoperative survival for patients undergoing resection of ICC. Interestingly, the RSI was not only a prognostic factor for early postoperative morbidity and mortality but also for long-term survival. This may be a result of an association between higher RSI values and tumor multifocality, which was identified as dependent risk factor for postoperative survival. Unlike other prominent scores, such as the recently validated MEGNA score, the RSI does not require postoperative data (e.g., histopathological staging) and thus can be of value not only for post- but also for preoperative therapeutic decision-making [14]. Of note, further well-known risk assessment scores, such as the FIB-4, ALBI, or Heidelberg score, were not further evaluated in the current study due to a lack of consistent preoperative monitoring of all required variables [33].

Due to the fact that the RSI focuses on the extent of hepatic resection, we also evaluated the influence of extrahepatic resections, such as additional hilar bile duct, vascular or multivisceral resections. Perhaps unsurprisingly, additional hilar bile duct resections significantly increased the incidence of severe postoperative complications, mainly as a result of insufficiency of the biliodigestive anastomosis and consecutive bile leakage. Extrahepatic or hilar bile duct resections are primarily associated with distal or perihilar cholangiocarcinoma. Thus, data on the rate and influence in patients with ICC is comparatively scarce. In a large meta-analysis on prognostic factors after resection of ICC, Mavros et al. reported a rate of extrahepatic bile duct resections (23%) comparable with the data presented in our study (19%). As observed in our uni- but not in multivariable analysis, some of the included studies demonstrated an association with inferior postoperative survival without commenting on the effects on postoperative morbidity [20]. Although definitions of (multi-)visceral resections are vague throughout the literature, sometimes including resections of major vessels or extrahepatic bile ducts, previous publications have emphasized that resection of adjacent organs results in inferior overall survival [10, 34]. In our study, multivisceral resection—defined as resection of extrahepatic tissue excluding extrahepatic bile ducts and major vessels—was identified as major predictor of inferior survival (with a median survival of only 4.96 months) in multivariable analysis. We therefore suggest critical evaluation of operability in case of pre- or intraoperatively suspected invasion of adjacent organs.

Lastly, histopathological factors, including positive resection margins, were evaluated as prognostic factors. A vast majority of available literature, including meta-analyses, states the importance of tumor-free resection margins to achieve long-term survival [10, 21, 35]. Surprisingly, positive resection margins did not have significant influence on postoperative survival, neither in uni- nor multivariable analysis. Of note, the width of the surgical margins was not analyzed in the present study since its relevance is generally discussed controversial: some recent publications pointed out that wide surgical margins (mostly defined as ≥ 10 mm) ensure better overall survival, whereas others reported conflicting results, especially with regard to lymph node–positive patients [36, 37]. Other known histopathological factors including high AJCC/UICC stages were confirmed as risk factors in univariable analysis, but only an advanced T stage was identified as an independent risk factor for survival in multivariable analysis in this study. As stated above, this is a result of the high rate of missing values regarding lymph node dissection and staging and consequentially AJCC/UICC classification, leading to an exclusion of the respective variables from multivariable analysis.

Although the role of lymph node metastases as a poor prognostic factor with concomitant high disease recurrence rates is undisputed, the benefit of simultaneous lymphadenectomy in patients with ICC has been a matter of great debate since the latest AJCC/UICC edition recommended harvesting at least six lymph nodes along the portal vein for proper staging [38–42]. In our patient series, we observed a slight increase of lymphadenectomies over the last three decades resulting in an overall lymph node dissection rate of 63%. Lymphadenectomy was associated with inferior survival in our patients as opposed to other studies in the past [43]. A systematic review and meta-analysis by Zhou et al. concluded that lymphadenectomy does not improve disease-free or overall survival but instead increases postoperative morbidity. Furthermore, in the case of lymph node positivity, lymph node dissection results in worse survival [44]. Kizy et al. even showed that surgical resection of lymph node positive ICC does not improve survival when compared with chemotherapy alone [45]. Others, however, have demonstrated better survival after lymph node dissection in case of lymph node negativity [46]. In summary, the positive effect of lymph node dissections for patient survival after resection of ICC remains highly questionable. However, keeping in mind innovations in medical treatment for cholangiocarcinoma, identification of lymph node–positive patients via lymph node dissection could prove crucial in determining further adjuvant therapeutic strategies in the future [47].

Major limitations of our study are missing information on disease recurrence and adjuvant therapy. Especially the latter seems important since recent meta-analyses by Rangarajan and Ma et al. have demonstrated the efficacy of postoperative adjuvant chemotherapy or radiochemotherapy for patients with positive resection margins, as well as lymph node infiltration after resection of biliary tract malignancies, and current guidelines on the matter clearly recommend adjuvant therapy upon resection [48–50]. However, Messina et al. reported opposing results in their meta-analysis and highlighted the increase of adverse events in patients undergoing adjuvant therapy after surgery [51]. Furthermore, our observations regarding the RSI are limited by the retrospective and monocentric nature of our study.

## Conclusion

Preoperative leukocytosis and the RSI are useful variables for preoperative risk stratification since they were identified as significant predictors for postoperative morbidity and mortality, respectively. Prospective clinical trials are now required to validate our findings.

## Data Availability

The datasets used and/or analyzed during the current study are available from the corresponding author on reasonable request.
